# Biomechanics of the press-fit phenomenon in dental implantology: an image-based finite element analysis

**DOI:** 10.1186/1746-160X-8-18

**Published:** 2012-05-29

**Authors:** Gianni Frisardi, Sandro Barone, Armando V Razionale, Alessandro Paoli, Flavio Frisardi, Antonio Tullio, Aurea Lumbau, Giacomo Chessa

**Affiliations:** 1 Epochè, Orofacial Pain Center, Nettuno, Rome, Italy; 2 Department of Prosthetic Rehabilitation, University of Sassari, Sassari, Italy; 3 Department of Mechanical, Nuclear and Production Engineering, University of Pisa, Pisa, Italy; 4 Department of Surgery, University of Sassari, Sassari, Italy

## Abstract

**Background:**

A fundamental pre-requisite for the clinical success in dental implant surgery is the fast and stable implant osseointegration. The press-fit phenomenon occurring at implant insertion induces biomechanical effects in the bone tissues, which ensure implant primary stability. In the field of dental surgery, the understanding of the key factors governing the osseointegration process still remains of utmost importance. A thorough analysis of the biomechanics of dental implantology requires a detailed knowledge of bone mechanical properties as well as an accurate definition of the jaw bone geometry.

**Methods:**

In this work, a CT image-based approach, combined with the Finite Element Method (FEM), has been used to investigate the effect of the drill size on the biomechanics of the dental implant technique. A very accurate model of the human mandible bone segment has been created by processing high resolution micro-CT image data. The press-fit phenomenon has been simulated by FE analyses for different common drill diameters (D_A_ = 2.8 mm, D_B_ = 3.3 mm, and D_C_ = 3.8 mm) with depth L = 12 mm. A virtual implant model has been assumed with a cylindrical geometry having height L = 11 mm and diameter D = 4 mm.

**Results:**

The maximum stresses calculated for drill diameters D_A_, D_B_ and D_C_ have been 12.31 GPa, 7.74 GPa and 4.52 GPa, respectively. High strain values have been measured in the cortical area for the models of diameters D_A_ and D_B_, while a uniform distribution has been observed for the model of diameter D_C_ . The maximum logarithmic strains, calculated in nonlinear analyses, have been ϵ = 2.46, 0.51 and 0.49 for the three models, respectively.

**Conclusions:**

This study introduces a very powerful, accurate and non-destructive methodology for investigating the effect of the drill size on the biomechanics of the dental implant technique.

Further studies could aim at understanding how different drill shapes can determine the optimal press-fit condition with an equally distributed preload on both the cortical and trabecular structure around the implant.

## Background

The use of dental implants has rapidly evolved since the advent of osseointegration, progressively replacing removable dentures in treatment of partially or completely edentulous patients. A fundamental pre-requisite for the clinical success in dental implant surgery is the fast and stable implant osseointegration.

The press-fit technique, which is adopted to ensure a primary stability condition for endosseous implants, requires the diameter of the hole drilled within the jaw bone to be smaller than the implant major diameter. The bone tissues are affected by a biomechanical phenomenon, which is characterised by the mechanical properties of bone, implant materials, difference between implant and hole diameters and surrounding bone morphology.

Recently, image-based approaches combined with Finite Element Analyses (FEA) have allowed effective stress–strain investigations in dental implantology. Dental implants can be virtually positioned within realistic models of human jaws reproduced from high definition CT image data with respect of the anatomical-physiological structures of bones. Worldwide, scientists have focused on this topic, especially to improve the success of endosseous implants [1-4]. A common goal is to understand the key factors of osseointegration processes following implant surgeries. Some researchers have investigated micro-displacements occurring at the bone-implant interface, while others studies have considered the load transfer at the interface to be more important in determining the correct mechanical stimulation of the osteoblasts, which are assumed to be responsible for bone tissue regeneration and the consequent osseointegration of the implant [5,6]. Generally, trabecular microstructures of bones are modelled as homogeneous entities with particular mechanical properties and contiguity assumed at implant-bone interfaces. The contiguity conditions do not allow relative motions between the parts generating a continuum of stress distribution at the interface, where stresses are generally concentrated. Limbert [7] has considered the trabecular microstructure of the mandible bone and the discontinuity at the implant-bone interface by a Finite Element Analysis. Further studies investigate the preload condition generated by the insertion of the abutment screw in the implant for different designs of the screw-abutment system [6]. Nowadays, the selection of drill diameter with respect to the implant geometrical configuration is still done without any scientifically tested criteria. Natali [8] has analysed the press-fit phenomenon occurring in oral implantology using the FE approach. In this study, the mandibular bone has been reconstructed by using CT data and attributing different mechanical properties in the cortical and trabecular regions.

However, a more detailed study should also consider the trabecular microstructure of real bone tissue and the contacts associated to the relative movement between implant and bone.

In this paper, an accurate model of human mandible bone segment is created processing high resolution micro-CT data by using image-based tools. The biomechanics of press-fit phenomena has been analysed by FE methods for different drill diameters.

## Methods

**Figure 1:**
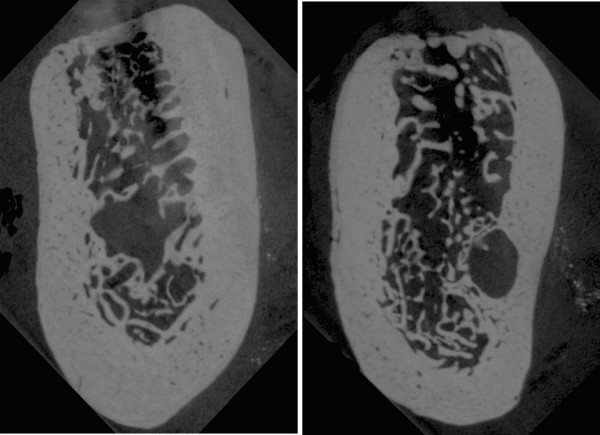
**Micro-CT scan data.** Micro-CT scan data of two different sections of the mandibular bone segment (35 μm resolution).

In this paper, edentulous bone segments of the right molar mandibular area have been acquired using a high resolution microtomography machine, the SkyScan micro-CT (SKYSCAN, Kartuizersweg, Kontich, Belgium). The sliced images have been generated with an isotropic resolution of 35 μm. Figure 1 shows the computed tomography images of two bone slices.

**Figure 2:**
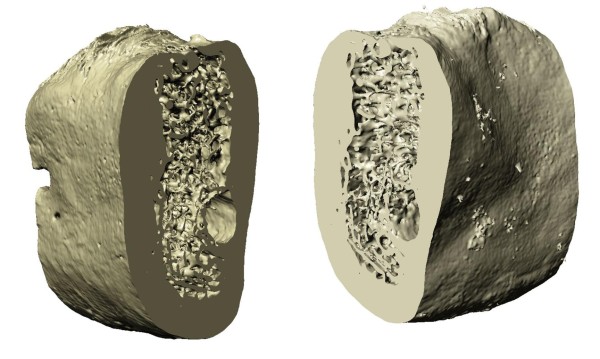
**Edentulous bone segment.** Different views of the edentulous bone segment generated in ScanIP.

The images from the micro CT have been processed to obtain the 3D models of the bone segments using the software ScanIP (Simpleware Ltd., Innovation Centre, Rennes Drive, Exeter, United Kingdom). 2D slice images have been processed by a methodology articulated in two main steps: segmentation and voxel creation. Initially, data have been converted to grey scale images. The region of interest has been isolated by thresholding the images and flood filling the masked area to avoid disconnected zones. The flood filling algorithm is based on a region-growing algorithm also known as *paint bucket*. The obtained segmented slices have been combined to produce isotropic voxels. The generated model is shown in Figure 2. The use of high resolution micro-CT data has allowed a detailed description of the trabecular bone microstructures with a voxel size of 130 μm.

**Figure 3:**
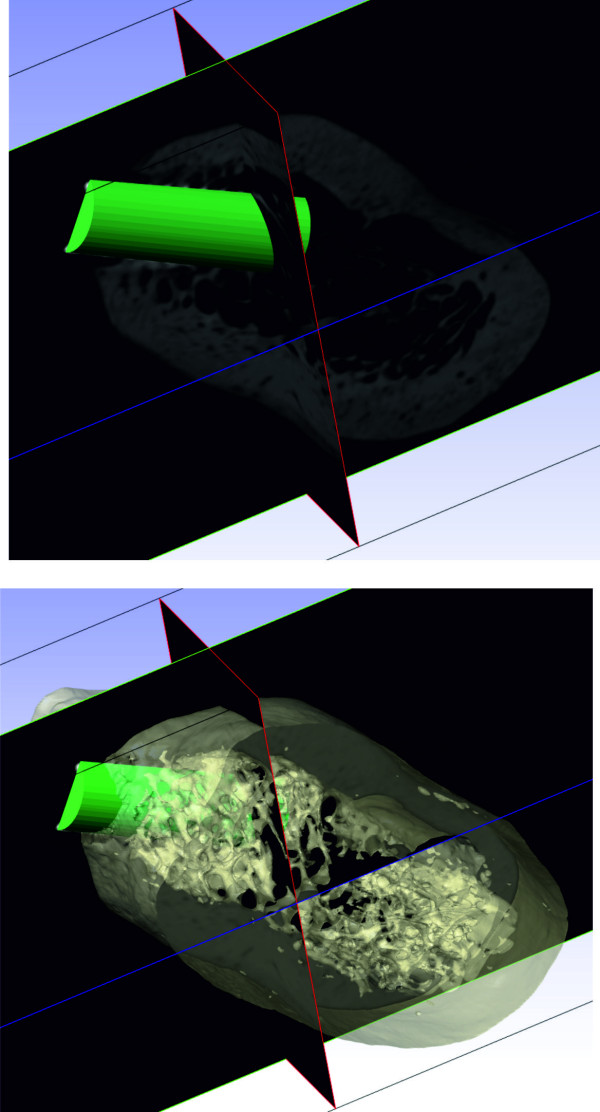
**Drill CAD model.** Positioning process of the drill CAD model.

**Figure 4:**
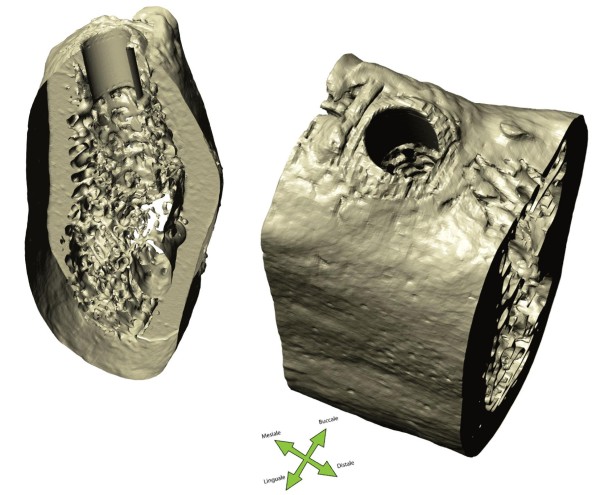
**Result of the virtual drilling process.** ScanIP re-imported model: (left) view through cut, (right) full volume.

The drill has been modelled assuming a cylindrical shape with different diameter values: D_A_ = 2.8 mm, D_B_ = 3.3 mm, and D_C_ = 3.8 mm and length L = 12 mm. The virtual implant model has been assumed with a cylindrical geometry having height L = 11 mm and diameter D = 4 mm. The drill models have been positioned into the bone model throughout the use of ScanCAD (Simpleware Ltd., Innovation Centre, Rennes Drive, Exeter, United Kingdom), which allows the placement to be driven by the corresponding 3D grey-scale micro-CT images and taking into account the bone structure (Figure 3). The result of the virtual drilling process is shown in Figure 4.

The edentulous drilled bone has been automatically meshed using the software ScanFE (Simpleware Ltd., Innovation Centre, Rennes Drive, Exeter, United Kingdom). The meshing process has been carried out using EVoMaCs approach [9], providing meshes of approximately 3 million elements (eight-node brick elements and four-node tetrahedral elements) and 900,000 nodes. The numerical analyses have been carried out by using the Comsol Multiphysics Analysis simulation software. The high 3D model accuracy has provided meshes free of any relevant volume/topology data loss.

The bone tissue has been modelled using an isotropic linear elastic material (Young’s modulus E = 15 GPa, Poisson’s ratio ν = 0.3 and density ρ = 731 mg/cm^3^) as proposed in literature [5,10]. The implant, typically made of Titanium (E = 115 GPa), has been modelled as a rigid body considering the huge difference between the bone and implant Young’s modulus and the effective reduction of the simulation computational cost.

The bone behaviour induced by inserting an implant with a major diameter into a smaller hole has been simulated using a contact algorithm (*Interference Fit*) based on the relative geometries, the material properties and the contact parameters. The assembly created after the implant positioning generates a volumetric overlapping between implant and bone shapes.

**Figure 5:**
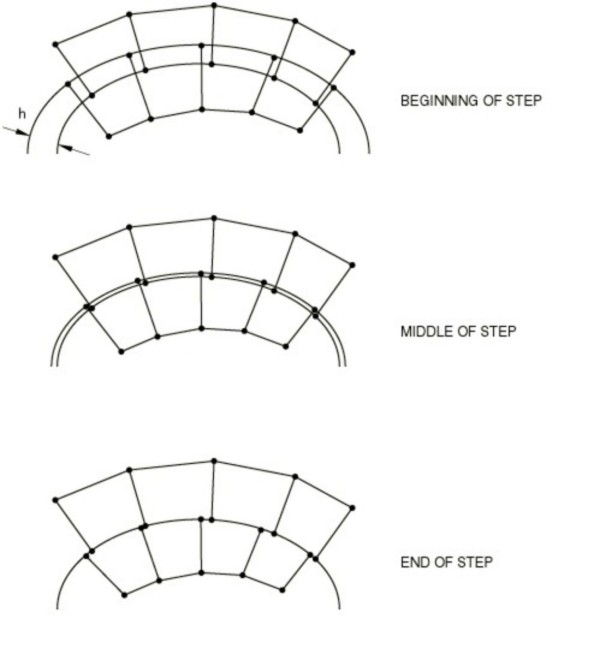
**Interference fit algorithm.** Contact constraint imposed at the bone-implant interface.

The contact constraint, imposed at each constraint location (bone-implant interface) to avoid overlapping zones, can be considered as the current penetration (h). Penetration exists when h is positive (Figure 5). This constraint can be updated by specifying an allowable interference (v), which is ramped down over the course of a step. The specified allowable interference modifies the contact constraint as h - v = 0.

The contact interaction property has been defined by a contact in the normal direction of the interaction (no penetration is allowed between the surfaces) and a *friction coefficient* μ = 0.2 in the tangential direction.

The lateral sides of the bone segment have been constrained in order to simulate symmetry conditions. The simulations have been run using Comsol Multiphysics Analysis and the Static step option. The static analysis approach has been used to analyse the stresses generated by the bone tissue stabilization following the press-fit phenomenon.

In accordance with the Declaration of Helsinki (Helsinki, 1964), written informed consent was obtained from the patient and the study protocol was approved by the local Ethics Committee. (University of Sassari no. 985)

## Results and discussion

**Figure 6:**
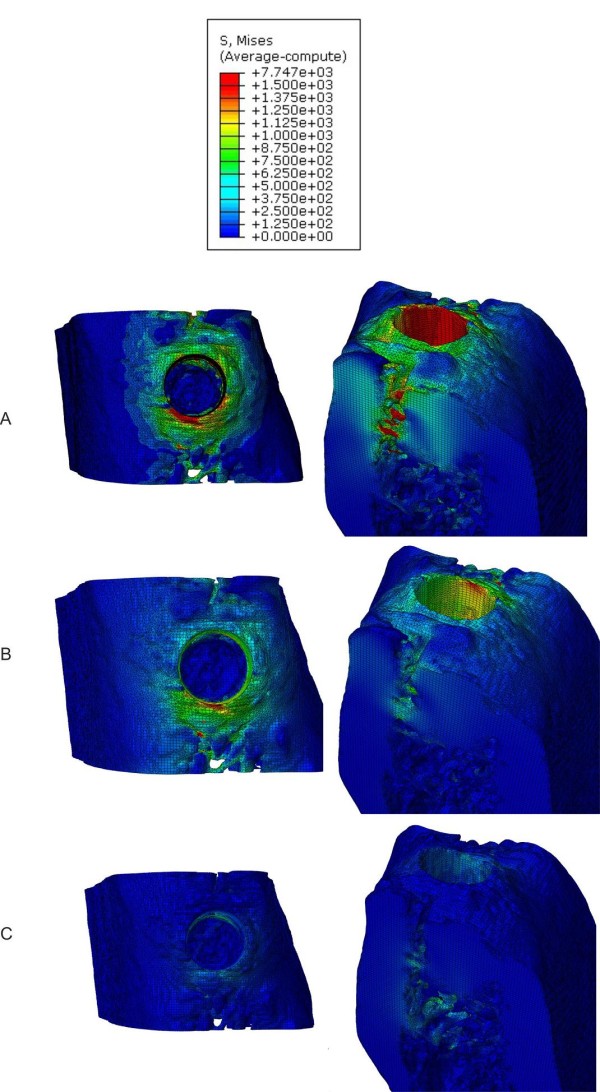
**Von Mises stress distributions.** Top view of the Von Mises stresses induced by bone tissue expansion for models A, B and C, respectively

**Figure 7:**
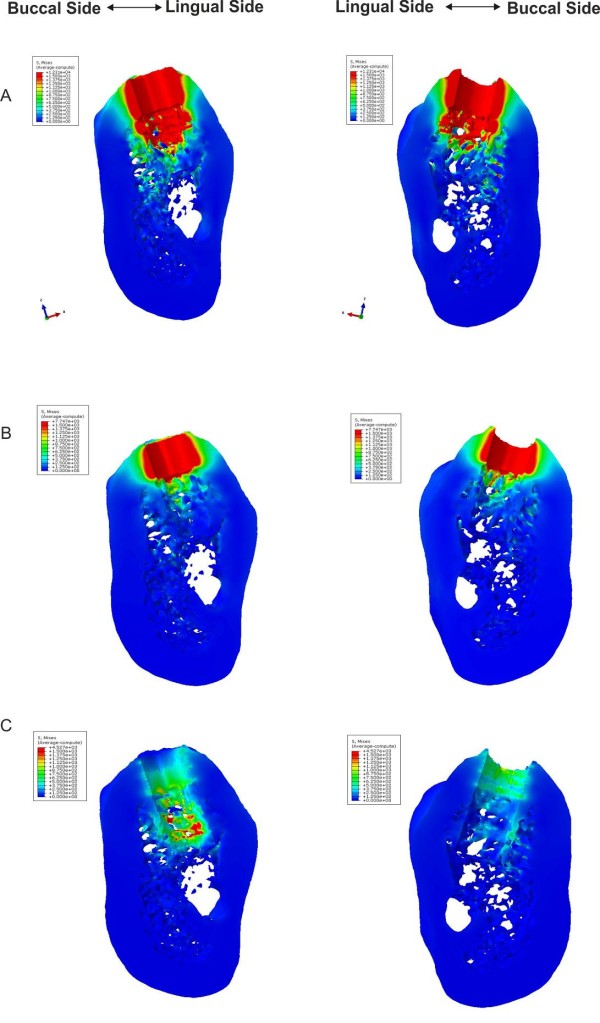
**Von Mises stresses distribution.** View of the Von Mises stresses distribution on opposite sides of the bucco-lingual virtual cut.

**Figure 8:**
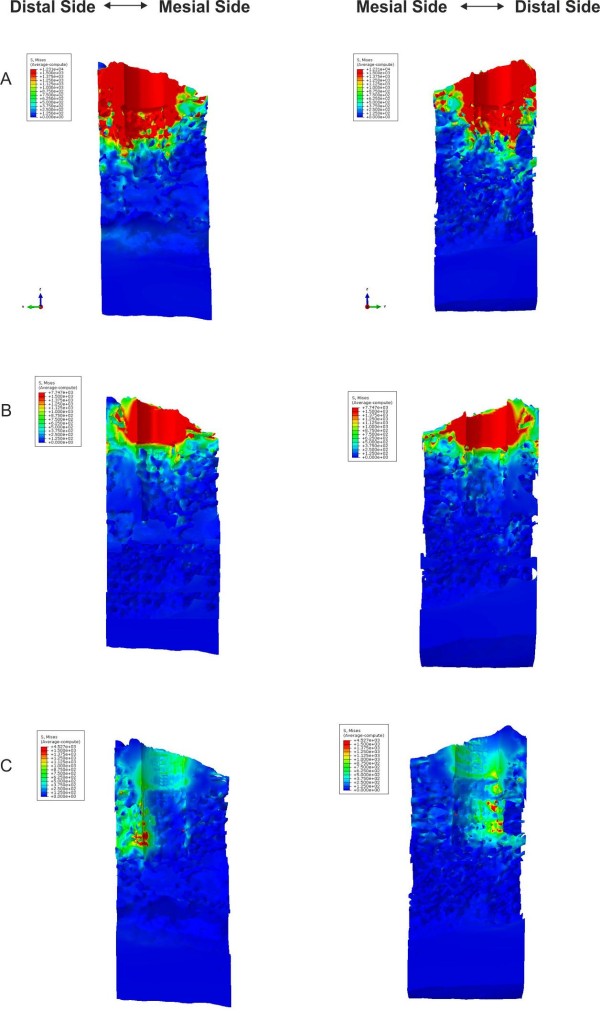
**Von Mises stresses distribution.** View of the Von Mises stresses distribution on opposite sides of the mesio-distal virtual cut.

The finite element analysis has been conducted for the three different drill diameter values. Figure 6 show the Von Mises stress distributions from a top view for models A, B and C, respectively. In Figures 7 and 8, the Von Mises stresses are presented along the bucco-lingual and mesio-distal planes. In models A and B, the press-fit phenomenon is almost completely supported by the cortical structure, though the implant is in contact with both the cortical and the trabecular bone. The stresses on the trabecular zone are minimal compared to those characterising the cortical area. In model C the stresses are equally distributed across the cortical and trabecular structure reducing the generation of critical and localised loads. A uniform stress distribution, as reported in model C, is the optimal condition for the osseointegration process allowing a distribution of bone cell stimulation all around the implant. High stress concentrations could determine bone cell necrosis or lack of homogeneous osteoblast stimulation, which are both possible causes of implant destabilisation. The maximum stresses in models A, B and C are 12.31 GPa, 7.74 GPa and 4.52 GPa, respectively.

**Figure 9:**
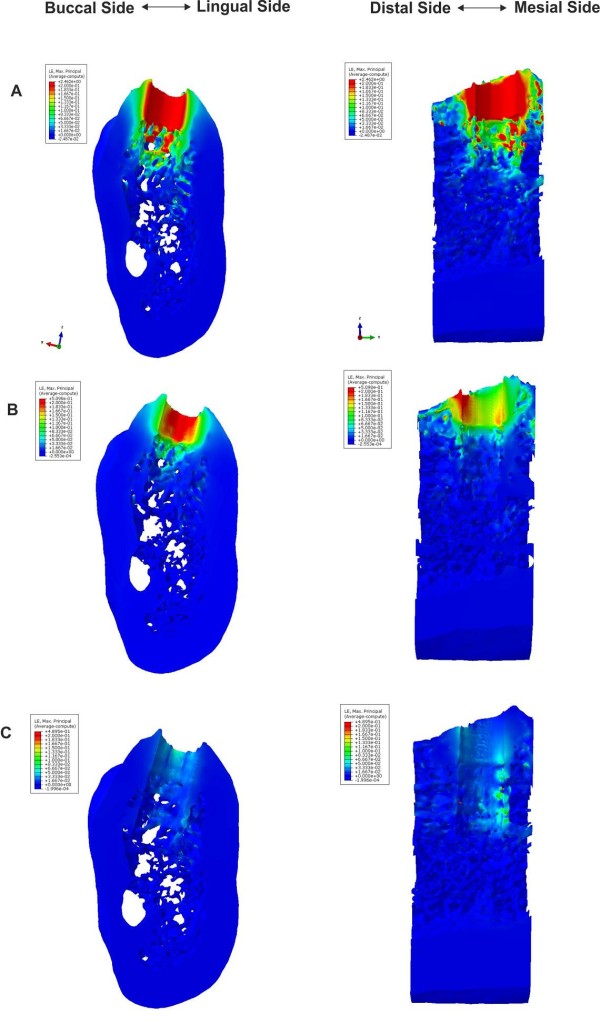
**Logarithmic strain distribution.** View of the logarithmic strain distributions on the virtual cut along the bucco-lingual and mesio-distal planes.

According to the stress distribution, high strain values are measured in the cortical area for the models A and B, and a uniform distribution is observed in model C. The maximum logarithmic strains, calculated in nonlinear analyses, are ϵ = 2.46, 0.51 and 0.49 for models A, B and C, respectively. The logarithmic strain distributions are presented in Figure 9 along the bucco-lingual and mesio-distal planes.

The press-fit phenomenon indicates a mechanical connection of two structures based on contact pressure. However, a more appropriate definition, “interference-fit”, could be used considering that each component mutually interferes to generate the deformation.

Generally, the actual viscoelastic behaviour of the bone tissue imply that the elastic recoil of bones decreases over time. The bone deformation and the elastic recoil depend on the density of bone tissue, and the cortical bone has a less viscoelastic behaviour than cancellous bone [11,12]. The results obtained in the present work demonstrate that the cortical bone is stressed and deformed significantly, even with moderate values of interference-fit. Figures 6,7 and 8 show higher von Mises values in the juxtacortical areas than in the bone marrow.

A viscoelastic behaviour also depends on the degree of initial elastic deformation of the bone when the press-fit is achieved. The “assembly strain” (i.e., the deformation of the bone tissue upon the introduction of the implant) has an effect on both the contact pressure between bone and implants (e.g. the initial press-fit) and the rate at which the press-fit is dissipated through deformation and elastic relaxation [13,14].

The results shown in Figures 7, 8, 9 indicate that the only press-fit value (pre-load) evenly distributing stresses caused by the bone-implant contact is generated with model C; whereas models A and B yield a uneven stress distributions. However, the stresses occurring in the model C may not yet be high enough to yield the necessary press-fit persisting in order to assure the primary stability. Once the initial press-fit is dissipated, the implant could move in the bone under load. This could suggest the use of an “undersized” approach that would only accentuate an elevated cortical bone-implant load using the techniques at our disposal.

The period between the initial press-fit and the healing or bone integration (intermediate period) is typically characterised by a relaxation of press-fit, which could be so fast to cause implant instability. The bone ingrowth, which leads to bone integration, is a very susceptible biophysical process, especially in the case of immediate loading of full arches where the absence of periodontal pressure receptors and the changes in the modulation of the trigeminal reflexes are no longer efficient.

The results of this study suggest the development of an alternative surgical procedure able to determine uniform stress and strain values between cortical and cancellous bone. This method should not be classified as a generic “interference-fit”, but as “differentiated interference-fit”.

A recent study [15] demonstrated that the undersized drilling technique, in addition to enhancing primary implant stability, might also achieve a translocation of bone particles. This biological phenomenon, comparable to the autologous bone graft, has a positive influence on the osteogenic response. However, this study shows that the difference between the implant diameter and the implant drill diameter is ∼ 0.6 mm corresponding approximately to model B.

According to the most recent studies focusing on primary stability [16] with an undersized approach [17], which is still equivalent to a press-fit of model B of this study, the occlusal load should also be considered, besides the obvious biophysical properties of the cortical bone and marrow. This does not correspond to a constant static and axial force, but to a dynamic, angular moment and extra-axial force with six degrees of freedom.

## Conclusions

This study introduces a powerful, accurate and non-destructive methodology for investigating the effect of the drill size on the biomechanics of the dental implant technique. Further studies could aim at understanding how different drill shapes can determine the optimal press-fit condition with an equally distributed pre-load on both the cortical and trabecular structure around the implant.

## Competing interests

The authors declare no competing interests.

## Authors’ contributions

GF, SB, AVR, AP, FF, AT, AL and GC participated to the conception and design of the work, to the acquisition of data, wrote the paper, participated in the analysis and interpretation of data and reviewed the manuscript. All the authors read and approved the final manuscript.

## References

[B1] Field C Li Q Li W Swain M Biomechanical Response in Mandibular Bone due to Mastication Loading on 3-Unit Fixed Partial Dentures J Dent Biomech 2010 2010 902537 20981154 10.4061/2010/902537 PMC2958459

[B2] Schwartz-Dabney CL Dechow PC Edentulation alters material properties of cortical bone in the human mandible J Dent Res 2002 81 9 613 617 10.1177/154405910208100907 12202642

[B3] Muhlberger G Svejda M Lottersberger C Emshoff R Putz R Kuhn V Mineralization density and apparent density in mandibular condyle bone Oral Surg Oral Med Oral Pathol Oral Radiol Endod 2009 107 4 573 579 10.1016/j.tripleo.2008.11.006 19168377

[B4] Al-Sukhun J Kelleway J Helenius M Development of a three-dimensional finite element model of a human mandible containing endosseous dental implants. I. Mathematical validation and experimental verification J Biomed Mater Res A 2007 80 1 234 246 17078048 10.1002/jbm.a.30894

[B5] Bonnet AS Postaire M Lipinski P Biomechanical study of mandible bone supporting a four-implant retained bridge: finite element analysis of the influence of bone anisotropy and foodstuff position Med Eng Phys 2009 31 7 806 815 10.1016/j.medengphy.2009.03.004 19395303

[B6] Park JK Choi JU Jeon YC Choi KS Jeong CM Effects of abutment screw coating on implant preload J Prosthodont 2010 19 6 458 464 10.1111/j.1532-849X.2010.00595.x 20456024

[B7] Limbert G van Lierde C Muraru OL Walboomers XF Frank M Hansson S Middleton J Jaecques S Trabecular bone strains around a dental implant and associated micromotions–a micro-CT-based three-dimensional finite element study J Biomech 2010 43 7 1251 1261 10.1016/j.jbiomech.2010.01.003 20170921

[B8] Natali AN Carniel EL Pavan PG Dental implants press fit phenomena: biomechanical analysis considering bone inelastic response Dent Mater 2009 25 5 573 581 10.1016/j.dental.2008.11.002 19128827

[B9] Young PG Beresford-West TB Coward SR Notarberardino B Walker B Abdul-Aziz A An efficient approach to converting three-dimensional image data into highly accurate computational models Philos Transact A Math Phys Eng Sci 2008 366 1878 3155 3173 10.1098/rsta.2008.0090 18573757

[B10] Lin CL Chang SH Chang WJ Kuo YC Factorial analysis of variables influencing mechanical characteristics of a single tooth implant placed in the maxilla using finite element analysis and the statistics-based Taguchi method Eur J Oral Sci 2007 115 5 408 416 10.1111/j.1600-0722.2007.00473.x 17850430

[B11] Lakes RS Katz JL Sternstein SS Viscoelastic properties of wet cortical bone–I. Torsional and biaxial studies J Biomech 1979 12 9 657 678 10.1016/0021-9290(79)90016-2 489634

[B12] Lakes RS Katz JL Viscoelastic properties of wet cortical bone–II. Relaxation mechanisms J Biomech 1979 12 9 679 687 10.1016/0021-9290(79)90017-4 489635

[B13] Brown CU Norman TL Kish VL Gruen TA Blaha JD Timedependent circumferential deformation of cortical bone upon internal radial loading J Biomech Eng 2002 124 4 456 461 10.1115/1.1488168 12188212

[B14] Shultz TR Blaha JD Gruen TA Norman TL Cortical bone viscoelasticity and fixation strength of press-fit femoral stems: finite element model J Biomech Eng 2006 128 1 7 12 10.1115/1.2133765 16532611

[B15] Tabassum A Walboomers XF Wolke JG Meijer GJ Jansen JA Bone particles and the undersized surgical technique J Dent Res 2010 89 6 581 586 10.1177/0022034510363263 20212102

[B16] Shalabi MM Wolke JG de Ruijter AJ Jansen JA Histological evaluation of oral implants inserted with different surgical techniques into the trabecular bone of goats Clin Oral Implants Res 2007 18 4 489 495 10.1111/j.1600-0501.2007.01362.x 17517059

[B17] Dhore CR Snel SJ Jacques SV Naert IE Walboomers XF Jansen JA In vitro osteogenic potential of bone debris resulting from placement of titanium screw-type implants Clin Oral Implants Res 2008 19 6 606 611 10.1111/j.1600-0501.2007.01519.x 18422985

